# GadgetArm—Automatic Grasp Generation and Manipulation of 4-DOF Robot Arm for Arbitrary Objects Through Reinforcement Learning

**DOI:** 10.3390/s20216183

**Published:** 2020-10-30

**Authors:** JoungMin Park, SangYoon Lee, JaeWoon Lee, Jumyung Um

**Affiliations:** Department of Industrial and Management Systems Engineering, Kyung Hee University, 1732 Deogyeong-daero, Yongin-si 17104, Korea; jmp@khu.ac.kr (J.P.); khutkddbs@khu.ac.kr (S.L.); wooni1019@khu.ac.kr (J.L.)

**Keywords:** reinforcement learning, robot gripper, point cloud, robot programming, object recognition

## Abstract

Automatic robot gripper system which involves the automated object recognition of work-in-process in production line is the key technology of the upcoming manufacturing facility achieving Industry 4.0. Automatic robot gripper enables the manufacturing system to be autonomous, self-recognized, and adaptable by using artificial intelligence of robot programming dealing with arbitrary shapes of work-in-processes. This paper specifically explores the chain of key technologies, such as 3D object recognition with CAD and point cloud data, reinforcement learning of robot arm, and customized 3D printed gripper, in order to enhance the intelligence of the robot controller system. And it also proposes the integration with 3D point cloud based object recognition and game-engine based reinforcement learning. The result of the prototype of the intelligent robot gripping system developed by the proposed method with a 4 degree-of-freedom robot arm is explained in this paper.

## 1. Introduction

Due to the acceleration to mass customization, automatic bin-picking has became crucial capability of modern manufacturing system. Lot size “one” production is upcoming market demands, as consumers’ needs vary, and so manufacturer started to customise their products and therefore requires ability to quickly manufacture various products. In particular, automatic robot gripper system which involves the automated object recognition of work-in-process in the production line is the key technology. Automatic robot gripper enables manufacturing system to be autonomous, self-recognized and adaptable by using artificial intelligence of tool-path generation dealing with arbitrary shapes of work-in-processes. But existing programming systems, such as Pick-n-Place, are not suitable for frequent change of the shape to be handled.

In terms of automation, Pick-n-Place task is one of the major important activity in the manufacturing system. Pick-n-Place of only a single object is relatively easy task because its object geometry is always identical in mass production line. But in the case of lot size “one” production [1], the Pick-n-Place becomes a difficult task, since it has to adapt many varieties of different shapes every time of grasping task. Most of manufacturing Pick-n-Place facilities are suitable just to standard shape of product geometry, which causes difficulty dealing with customised products.

It is not straightforward for robots to completely adapt their control program of Pick-n-Place tasks to arbitrary product shapes without humans’ inputs. For this reason, many factories still produce their robot arm paths by on-line programming of material handling. Most well known method of this way is using ‘teach pendant’. It is efficient for picking identical shape by manual programming because the robot program does not change after a robot expert made it once. But, it is an inefficient method when responding to lot size “one” production having to reprogram the robot path every single time. And also the quality of the path generation could vary according to the worker. Implementing intelligence and flexibility that makes the robot smart, could solve the above-stated problems with reasonable investment which will lead to profitability.

**Figure 1:**
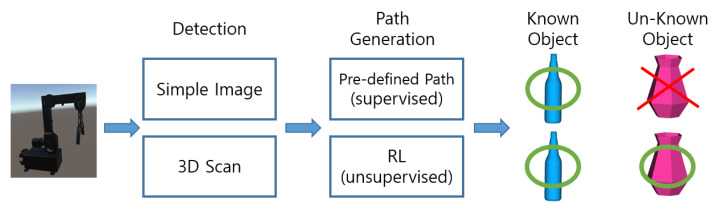
Simple process of commercial robot and GadgetArm.

This paper suggests using RL (reinforcement learning) for automated robot arm path generation and deep learning for object classification to grasp various arbitrary object. Legacy commercial system of automatic bin-picking uses 2D based image detection algorithm to detect the orientation and location of target product and predefined robot path after long and large training are applied. But the authors propose the integration with PointNet object recognition algorithm and game-engine (Unity) in RL. Automatic object recognition, reinforcement learning of robot arm are the main technologies of the proposed method. The proposed method reduces the effort to prepare training data and the expertise to establish predefined program as Figure 1. By implementing this method, difficult Pick-n-Place task could be easily solved with low cost device. This paper specifically explores the chain of key technologies, such as 3D object recognition with point cloud data, RL of robot arm and gripper, and a customised 3D printed gripper, in order to enhance the intelligence of the robot controller system which can lead to highly mass customised production line.

In Section 2, related and existing models are introduced, such as fundamental researches to realize autonomous robot programming. In Section 3, describes the overall intelligent robot controller architecture of automatic robot gripper with more detailed explanation composed with object detection, automatic tool-path generation, and robot programming. Then in Section 4 the result of the prototype of the intelligence robot gripping system, developed by the proposed method with a 4 degree-of-freedom robot arm is explained. Section 5 discusses the comparison and improvement with legacy robot programming(teach pendant) method and proposes intelligent gripper system method “GadgetArm”. Conclusions are briefly given in Section 6.

## 2. Literature Survey

In this section, previous related methodologies for robot arm grasping are introduced by dividing them in three parts. First, a method for robot arm to recognize object recognition by 2D data format to 3D data format is presented. Then various ways of training the robot arm to grasp the object properly, automatically and combining them to the robot programming are introduced. Finally a summary and opportunities of the related work are discussed.

### 2.1. Object Recognition

In the past there were many approaches to deal with robot arm’s limitation, grasping unknown object reliance to humans vision system with online programming. Traditionally used 2D contour image data for object recognition in grasping [2,3,4].

Development of a camera that captures depth data made it convenient to earn 3D data format, which would provide more precise information compared to 2D data format. Also, improvement of DL (deep learning) made it possible to use 3D data as input in object recognition tasks by various DL algorithms. Most well known and used 3D data formats are Voxel and PCD (Point Cloud Data). 3D ShapeNet [5] and VoxNet [6] uses Voxel data format to classify object. But since voxel data format increases the data size and obscure recognizing the essence of the shape, PointNet [7] used PCD for object classification. The advantage of the PCD is that since it is the raw data of the object, it is accurate and has acceptable data size. In the same way, Wang et al. proposed a novel global PCD descriptor to improve robot grasping operation by using object recognition and pose estimation [8]. And Ji et al. combined real-time grasp control with vision data in order to achieve reliable and accurate object grasping even though operation in a cluttered scene [9].

### 2.2. Existing Platforms of Reinforcement Learning

Using RL in various fields has been successful. This is due to well made simulation software tools and well described by Erez et al. [10]. These tools are widely used especially in robotics. Johns et al. used Dart simulation physics engine with CNN image processing to grasp object with parallel gripper [11]. Also with most popular physics engine Mujoco integrated with OpenAI Gym, Plappert et al. tested robotic task such as reaching, pushing, sliding and Pick-n-Place task [12] and Wang, C. et al. trained Husky Dual UR5 robot for mobile manipulation by RL integrated with camera for object pose estimation [13]. But these simulation software tools are difficult to build complex simulation environment. So for alternative, games based simulation engine Unity is used for RL task, since it is more convenient building complex simulation environment. Mousavian et al. used game engine Unity, integrated with physics engine Flex to generate successful grasp for training data [14]. Advantages of Unity simulation platform are easy to learn and have sufficient physical complexity, also provide compelling cognitive challenges, and support dynamic multi-agent interaction [15].

### 2.3. Automatic Robot Programming in Smart Factory

Demands of gripping arbitrary objects in an industrial environment have also been discussed in papers for the flexibility of smart factories. Reddy et al. stressed the importance of universal gripper, more like a human hand, to avoid changing the gripper for each part. The human-like hand would make the gripper commonly suited for use in arbitrary areas from household chores to variable industrial tasks [16]. Birglen and Schlicht aimed at presenting characteristics of commonly used impactive grippers to pneumatic and parallel finger movement grippers in terms of performance index referred to as a C-factor which including stroke, force, and weight specifications. With these statistical analysis data, they could figure out that even though the variety of grippers on the market is large, almost all of them share similar characteristics that are used in the industrial environment [17]. Zhang et al. pointed out the complication of existing self-calibration accompanied by camera calibration, corner detection and laser tracking. To solve the problems, they introduced inertial measurement unit and position sensor to obtain the robot poses by Quaternion algorithm and Kalman filters. After the estimation, extended Kalman filter was used to carry out kinematics identification to improve the accuracy of robot gripping [18]. In the same way, automatic grasp generation has been developed by several researchers to improve the intelligence of robot programming. Kraft et al. suggested methods of automatic grasp generation to replace manual grasp selection with a bin-picking process to reduce cost [19]. To reduce the cycle time of the process planning, off-line programming method was implemented by calculating inverse kinematic automatically [20]. Weigand et al. applied a Runge-Kutta neural network as a neural adaptive control in order to reduce gearbox errors on industrial robots [21]. Also, recently, to avoid slip Muthusamy et al. proposed the robot system to grasp an object with slip detection by using computer vision though real-time direct robot programming [22].

### 2.4. Summary and Opportunities

The conclusions we can draw after reviewing the literature are:

1. The increase of the rate of object detection has possibility to replace the capability of human observation which is the guide of robot position in legacy robot programming.
2. Traditional programming methods, such as legacy on/off-line programming, are limited in relying on human expertise rather than improving the automation of mass customization.
3. Lot size “one” needs the high flexibility to handle all different shapes of work-in-processes.

From these conclusions it appears that there is opportunity to motivate the autonomous robot control of different shapes. As well as there being a clear need for alternative technological solution in this area, the series of intelligent robot programming will ensure that the new development will provide benefits in the personalised material handling.

## 3. Conceptual Model of Automatic Grasp Generation

### 3.1. Overall Architecture

Typically legacy robot gripping applies without automatic grasp generation. Humans can detect a product and think of a grasp strategy without difficulties while a robot needs programming task before picking objects. Typical robot programming is the iteration of eye-observation and path-adjustment on teach pendant. Simple picking is reasonable to manual handling because of small benefit of robot gripping but the high cost of robot systems. Human expertise is required even though using off-line programming finds an optimised path among the alternatives generated automatically. This means that gripping task depends on the knowledge and the experience of human operators in both methods.

For this reason, automatic grasp generation proposed in this paper is a low-cost system dealing with arbitrary shapes. The main considerations of automatic grasp generation are (1) pre-training from CAD data and (2) one-shot handling without programming iteration like teach pendant. Pre-training is finished before work-in-process arrives in the production line. All try-and-error finding optimal path are finished in virtual reality. Object detection helps for searching the optimal path of the object arrived in production line. By pre-training and object detection, one-shot handling without any iterations can be realized. And the automatic grasp generation keeps seamless performance of grasping accuracy even though human operator replaces to another operator.

To develop the automatic grasp generation system, the overall architecture consists of object recognition, automatic positioning, and integrated robotic system. This chain of components reduces manual operation and allows labour to be used elsewhere. Whenever the product shape manipulated by robot arm is changed, either iterative programming or robot program needs to generate new robot path again.

**Figure 2:**
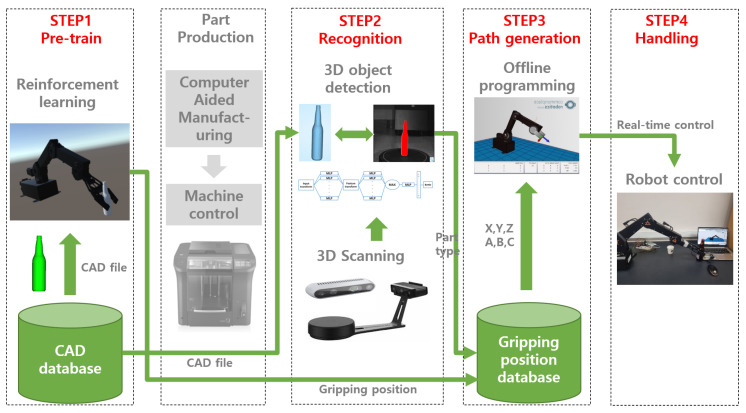
Overall Process of automatic grasping programming.

An automatic robot gripper system for autonomous manufacturing systems requires two technologies which are automated object recognition, path-finding and grasping for a full automation system. This paper suggests an innovative way to satisfy the stated requirement by using a depth camera for 3D scanning and a video game engine (Unity) for RL. The overall procedure is explained briefly in Figure 2.

1. STEP1: After the product designer uploads new CAD file of new part shape on manufacturing database, the file is imported into the virtual reality of RL. The virtual reality consists of two training environments of two-step RL. First step is to find the gripping position. Second step is to find the path of the robot arm to reach the gripping position. Two-step RL and virtual reality were made by Unity ml-agents. Each gripping position and robot path optimised are transmitted into manufacturing database via SQL query. Then, CAD data is also transmitted into production line. Factory starts to produce the new part as the physical object of new part.
2. STEP2: After part production of a new part shape is laid down on the working table of a material handling facility surrounded by a 3D scanner. 3D scanner traces the reflection curves of scanning laser and converts the signals into point cloud file. The PCD is input data of 3d object detection. PCD is stored in the file system of PointNet algorithm.
3. STEP3: When 3D scanning is done, PCD is utilised to find the part type which just arrived by using PointNet, one of 3D object detection algorithms. Based on the PCD, the part shape placed on working table is detected by classification algorithm of PointNet. The output of this step is classifying which type of part shape is arrived in production line. The part type found is sent into next step.
4. STEP4: The part type classified by PointNet and the gripping position returned from the database are the input of this step. First task of this step is to send a query of part type detected by PointNet to manufacturing database where gripping positions of all part shapes are stored. Robot controller manipulates the gripper along the gripping position returned from database. The chain of these steps makes the robot control adaptive to arbitrary part shape.

### 3.2. Recognition by Point Cloud

**Figure 3:**
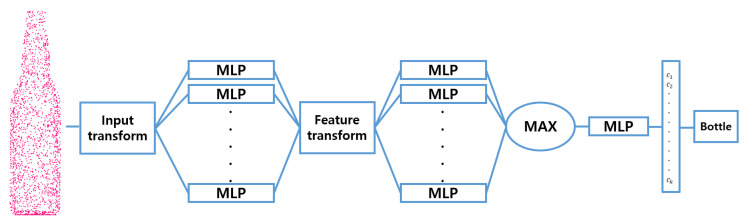
PointNet Architecture for Classification.

An object recognition task is automated by using 3D data in order to improve classification accuracy and data handling. Proposed architecture apply PointNet [7] architecture (Figure 3) to point cloud classification task with CAD data and 3D scanned data.

Object recognition tasks were traditionally done by methods such as RFID tag or simply by the human eye. Tagging RFID object could be the easiest way for automating object recognition. But tag needs to be attached to each object and this causes operation cost which could not be ignored. The most primitive way, recognized by the human eye, is used in a teach pendant. RFID tag and human recognition are expensive methods in the case of mass customization. Automatic process is required to follow market change.

To make it automatic, the authors propose the integration of a 3D scanner and a deep learning algorithm. Using 3D data improves classification accuracy and data handling. Improvement of 3D camera makes it possible to collect depth data and extract point cloud data. Using 3D data improves the accuracy of the classification of arbitrary objects rather than using 2D image. Only by the 2D data format, it is hard to recognize a 3D geometry accurately due to limited viewpoint. Most known 3D data used for classification task are Voxel format and Point cloud format. But when using voxel data format, data becomes unnecessarily large because it approximates cubic representation of the object. Earning richness of geometry information and avoiding complexities that make the calculation faster is possible by directly taking point cloud as input.

In this paper, accuracy for classification task was considered as an important factor to enhance the performance of the system. For this reason, choosing PCD as an input format was necessary, since it could provide both enough geometry data and reasonable data size for the overall process. The authors used PointNet architecture for point cloud classification task. It is a simple and robust deep learning algorithm for classifying and segmenting object using directly PCD as input. As PointNet architecture receives PCD as its input, it is used as the main algorithm for classifying object.

There are mainly two methods for collecting PCD.

The first way of collecting PCD is from the CAD file. Even though there is tolerance between design shape and manufactured shape, the existence of CAD data is reliable input of object detection. CAD file is shared by product data management system commonly used by most manufacturing industry. By using CAD file, for example .obj format, it can easily be changed to point cloud format and extract point cloud. Also it is possible to change the density of the point cloud. High density of points means extracting high number of points from the object. Extracting high density of point can give more accurate geometry information, but too much can cause degradation of computing speed of classification algorithm. To make the algorithm fast enough, but still has enough geometry information, 2048 points are chosen from the point with poisson-sampling method for point sampling in order to sample uniformly.

The second way is 3D scanning an object using depth camera. 3D scanning takes longer time compared to a fixed depth camera in case of collecting data. But when the camera is fixed, it could collect imperfect data, only about half of the geometrical data, since the opposite part is not being shown. The object detection task by 3D scanning finishes in a short time which is allowable in production line. The proposed system also uses only single algorithm for object detection. Using an another algorithm makes the system more complicated and reduces the speed for classification task.

A test dataset is generated from CAD file and 3D scanning. CAD file is labelled by the designer and converted into training data of PointNet. 3D scanning data is used for the input during operation. PointNet compares 3D scanning data with the training data and returns the label indicating product model.

### 3.3. Reinforcement Learning in Unity Ml-Agents

The proposed automatic robot programming system employs the RL in order to generate the gripping position of each part shape. The virtual reality of RL consists of robot arm, gripper and part shape to be manipulated. It provides the environment where a machine learning algorithm attempts all actions that robot arm can do. For this paper ml-agents [23] was used, an RL toolkit provided by Unity Technologies, to make robot arm learn how to grasp the object.

A high DOF (Degree of freedom) of RL has a relatively high possibility of going in the wrong direction during training. The target robot arm has 6 behaviours which are 4-DOF of robot arm, gripping action, and application of gravity. Before the behaviour of the gripper gets stable, part shape might be hit by unexpected gripper movement during training mode. Hitting objects cause the part shape to fly away and lead to rapid decline of the learning rate. Limiting DOF in time is required for avoiding an unexpected learning direction.

For this reason, training environments are separated into two pieces. The proposed RL is composed of gripping training and robot arm training. Separating the training into two parts reduces the running time to achieve finding optimised gripping position. Gripping training has 2-DOF of gripping action and gravity application. And robot arm training controls 4-DOF rotations.

**Figure 4:**
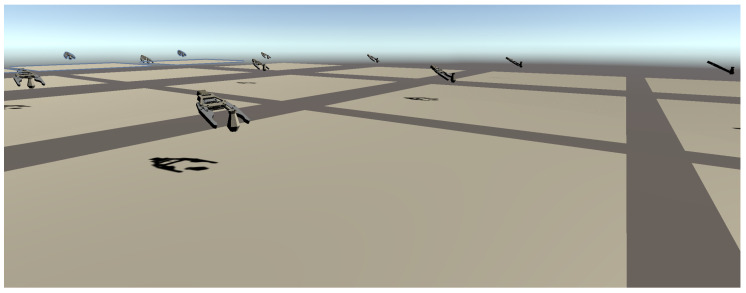
Using multi-agents for saving learning time.

In this work, the PPO (Proximal Policy Optimization) [24] algorithm was used. PPO is one of the policy gradient method algorithms. PPO is easier to implement and has more ease of sample complexity than the previous policy gradient algorithm TPRO (Trust Region Policy Optimisation) [25]. PPO has also displayed the best performance on continuous control tasks. One of the advantages of ml-agents is making multi-agents at the same time like in Figure 4. This function saves time for RL since it could test various grasp positions simultaneously. This way we can get the gripper’s position where the gripper can grasp the object successfully. Then the gripper’s position is sent to the robot offline programming software to move the robot arm at grasp position in real.

**Figure 5:**
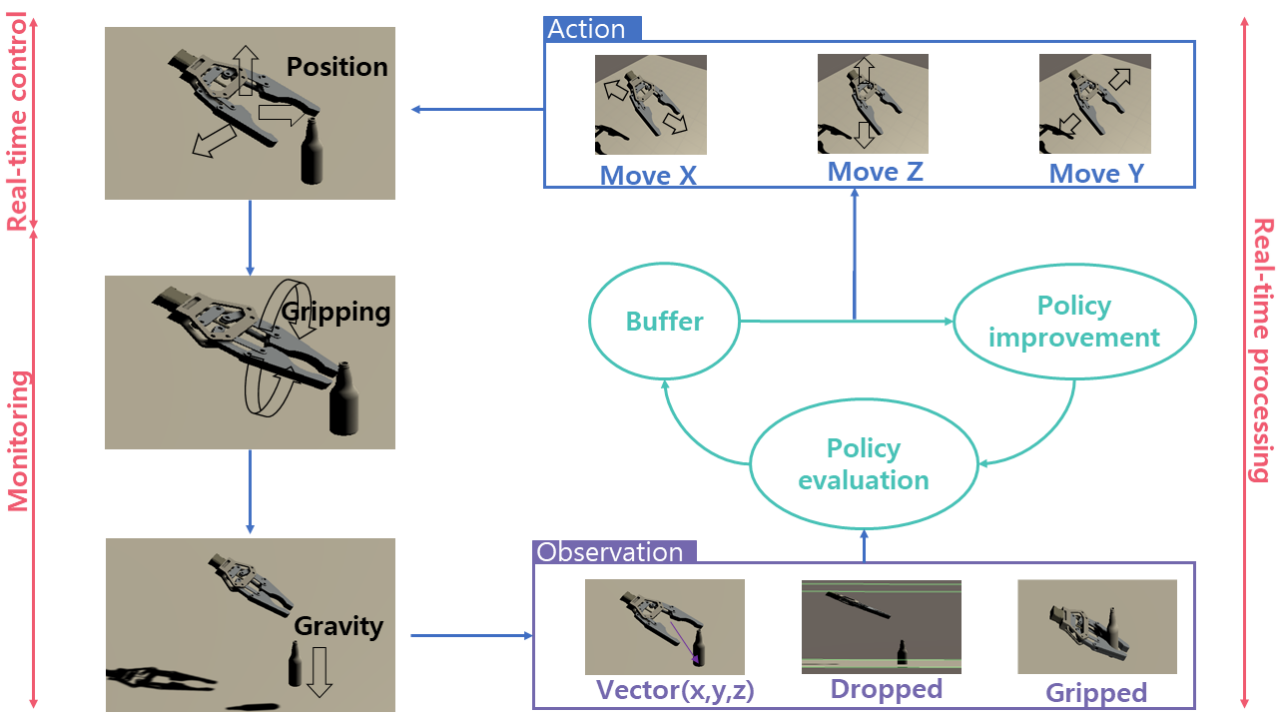
Training process of Reinforcement Learning.

Basic components of RL are agent, observation, action and reward. In this RL, the agent is the gripper and observation, action, reward is generated by the Unity environment shown in Figure 5. First, gripper moves along X,Y,Z axis. During RL the agent observes distance of gripper to bottle as Vector3 value(x,y,z) frame by frame. Next, gripper runs grasping motion and after applying gravity to the object to check if it has grasp well or not. If an object is dropped, the agent gets minus value reward, and if gripped correctly it gets plus value reward.

### 3.4. Robot Programming & Gripper

Robot programming methods can be divided into two categories—online programming, commonly used with teach pendant connected to the robot arm via a cable or lead through method, and off-line programming. Although online programming is easily suitable for all types, improving off-line programming is necessary to realize industrial 4.0 for the reason that the suitability of online programming decreases as the industrial processes become complex. Furthermore, from an economical perspective, off-line programming only could be justified for large volumes of production in big enterprises. However, by cooperating with RL in Unity ml-agents, this method can break through the limitations compared to commonly used robot programming using augmented reality which combines the features of online and off-line programming to make off-line programming more interactive and flexible.

## 4. Prototype Implementation

### 4.1. Target Objects

**Figure 6:**
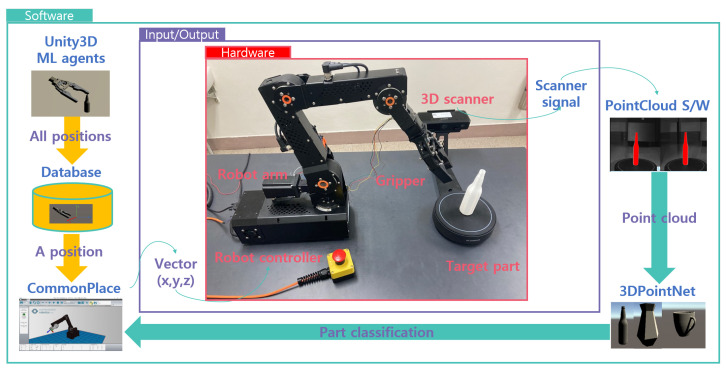
Prototype simulation environment.

This section is mainly focused on introducing the prototype of the GadgetArm and how it is implemented. As mentioned above, this paper is suggesting a simple and reasonable method for automatic grasping. Any object that can be classified by PointNet can be our target object. In this work, a bottle was chosen as the main target object because it could be easily obtained and has reasonable size considering the size of the gripper. For the test environment, the gripper was made using a 3D printer, which modified the open source design to fit the robot. Also modified gripper was controlled by Arduino-UNO board for grasping and connected with IgUS Robolink RL-DCi-4-DOF. The robot arm could reach 510 mm and payload 1000 g with 4 Degree of freedom. For vision task to extract PCD, Intel RealSense D435 was used as fixed depth camera and for 3D scanning task, Shining 3D scanner was used. Ideal prototype simulation environment are shown in Figure 6. Proposed architecture was implemented within the test environment where Windows 10 PC equips NVIDIA GTX 1060 GPU and 16 GB memory.

### 4.2. Implementation of Pointnet

**Figure 7:**
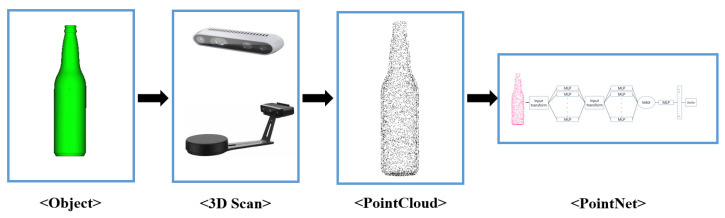
Process of recognition task.

**Figure 8:**
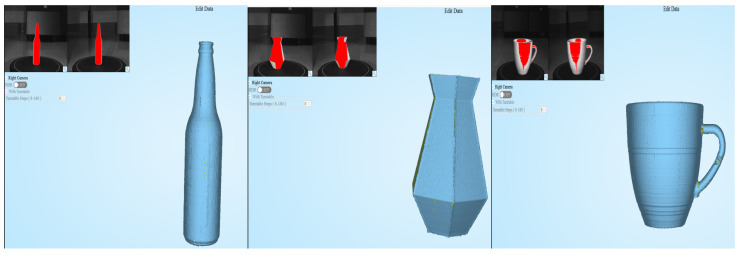
3D scanned result of bottle, vase and cup.

The process of the recognition task is explained in Figure 7. As mentioned above, a bottle, vase and cup were selected as the target object. If there exists a CAD file, it could be easily converted to PCD format by various open software. In case, where no CAD file exist, it could be scanned by 3D scanner to get the PCD. For experiment, even though existed an actual size CAD file, printed with 3D printer and 3D scanned the actual bottle for testing. For both two cases, CAD file converted to PCD and 3d scanned PCD, 2048 points were sampled by poisson sampling method using PCU library [26]. Training PCD set was based on ModelNet40 [5] dataset. For custom target object, in this case bottle, vase and cup, CAD data was earned from web crawling which is 1 to 1 scale to the real object. To test 3D scanning method, printed the object with the 3D printer and after then performed scanning as Figure 8. Shining 3D EinScan-Se was used for scanning. Another important process left is scaling the size of the PCD by zero-mean and normalizing into a unit sphere for effective learning. Finally, by the algorithm based on PointNet which classifies up to 40 objects, classifies which class it belongs to. To test the performance of the PointNet algorithm, PCD earned from both CAD data and 3D scanning were used for each object type. The algorithm classified well on both cases on bottle and vase. But the cup PCD from CAD data was not able to classify correctly, even though PCD from 3D scanning classified perfectly. According to the classification result, the data of the object (CAD data) information is delivered to the Unity environment to start RL.

### 4.3. Reinforcement Learning

**Figure 9:**
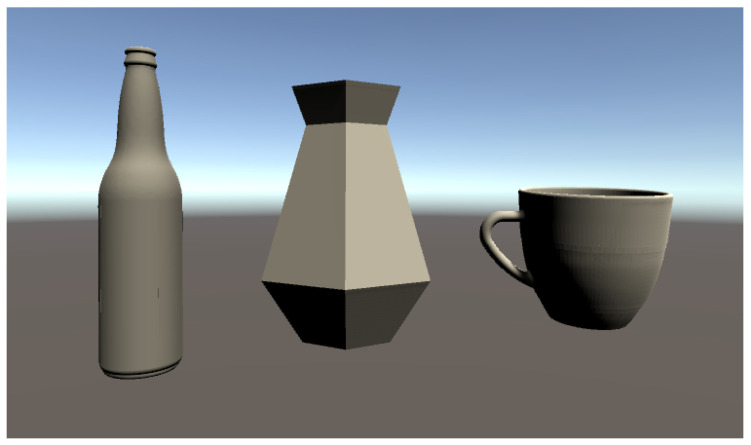
3D objects in Unity.

Among the objects in ModelNet40, we selected three objects for RL—bottle, vase, and cup shown in Figure 9 as the ones that the gripper can grasp in reality. The version of RL environment is Python 3.7, Tensorflow 2.3.0, ml-agents 0.18.1. And we used the following PPO hyper-parameters Table 1.

Training model was trained on a continuous environment and the gripper was set able to move XYZ axis and rotate only by B axis since the tested robot was 4-DOF. Final gripping position derived from gripping training is the input of robot path training in two-step RL. Robot path generators are also provided by on-line/off-line program system of commercial robot system. This section explains the detail of gripping training because robot path training is replaceable to legacy robot system.

For reward progress it was divided into 3-steps—(1) Gripper movement stage, (2) Gripping stage, (3) Apply gravity. Reward was given at each stage for a step-wise learning. The gripper observes the distance to the object and learns to get closer, otherwise it gets negative reward. Colliders which can detect collision with others were added to both sides of the gripper and got positive reward only if the object and the both side of gripper were tagged at the same time. After the gripping stage, gravity was applied to the object to find out whether the object was grasped properly. To train the gripper to keep holding the object, two box colliders were added, one at the bottom floor and one at the above the gripper, so in case of object drops or object flies away it ends episode and is set to get negative reward. The data flow of gripping training is depicted in Figure 5.

**Figure 10:**
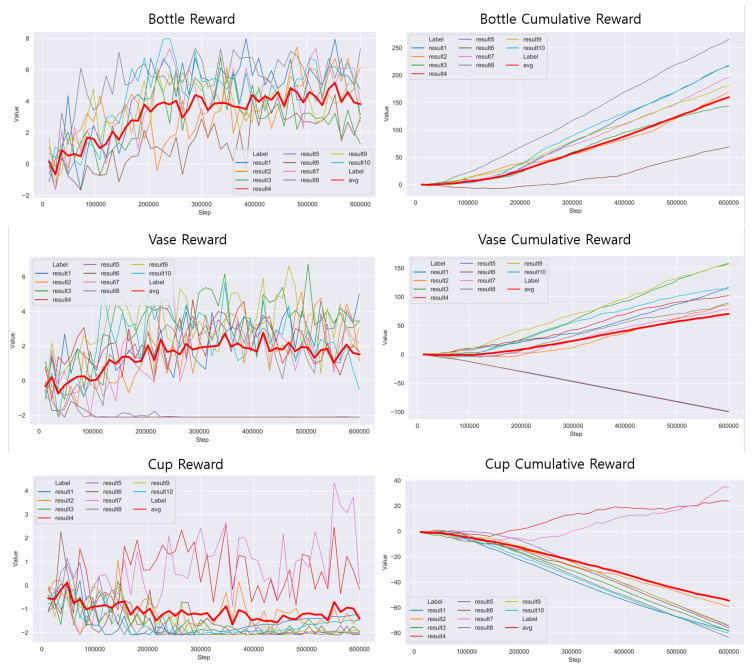
Results of reinforcement learning.

The reward and cumulative reward graphs are shown in Figure 10. We trained three objects for 500,000 steps and repeated it 10 times independently. The gripper is trained more stable in bottle than other objects. This result is because of constant cylinder shape of bottle. Round shape fits well with the shape of our gripper and gripper can grasp anywhere more successfully than a cup which is asymmetrical.

**Figure 11:**
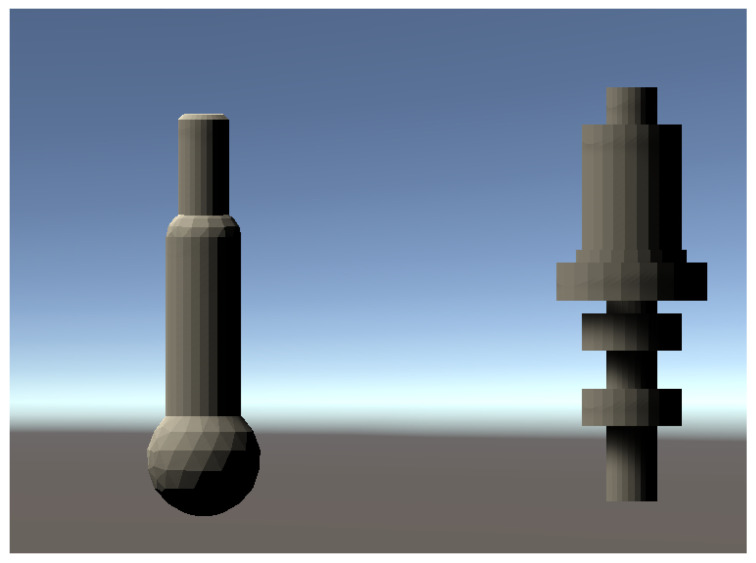
Industrial parts in Unity.

**Figure 12:**
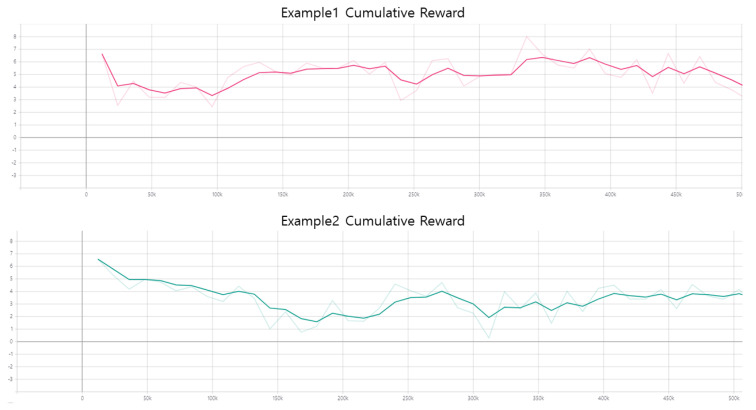
Results of reinforcement learning for Industrial parts.

With the consideration of industrial applications, we added two turning machining examples as the industrial experiments. Industrial parts are shown in Figure 11. All the turning examples are tested with standing position. because typical production line brings the parts on pallet system. The cumulative reward graphs of GadgetArm reinforcement learning are shown in Figure 12. We trained two objects in the same conditions to the above experiment. Two graphs always have a value greater than 0 and keep high average rate. It means learning results are stable rapidly and do not decline easily. Even though simple shape parts, industrial robots need to fine control to have stable gripping program of robot arm. The proposed methods let operators not to consider about detail of robot motion after the parts are located in fixed pallet. This means robot arm can learn well to grasp industrial parts too.

### 4.4. Robot Programming

Most robot arms have their own programming language based on their software and users can improve by themselves to drive the robot arms. Igus Robolink robot arm was used with Commonplace CPRog programming environment to drive the robot arm, and XML files were used to control it wirelessly through TCP/IP protocol. Therefore whenever creating an optimal path in Unity, a 3D simulation environment, it was necessary to fit this data into an XML format. It was automatically converted by Unity to XML format and the experiment was conducted by using the paths.

Robot programming to grasp the object used the optimal coordinates obtained by RL in Unity. This coordinate was used in the Igus Robolink software to move the robot arm to the coordinate in reality. However, in Unity, the robot arm joints deflection due to the mechanical vibration and the influence of gravity is not considered, so the process of matching the reference using the program was additionally required. Therefore, in order to minimize vibration, the speed of movement of the robot arm was slowed down to optimize, and the amount of change in the z-axis (vertical axis of ground plate) was considered as the optimized coordinate through multiple iterations.

## 5. Discussion and Conclude Remarks

**Figure 13:**
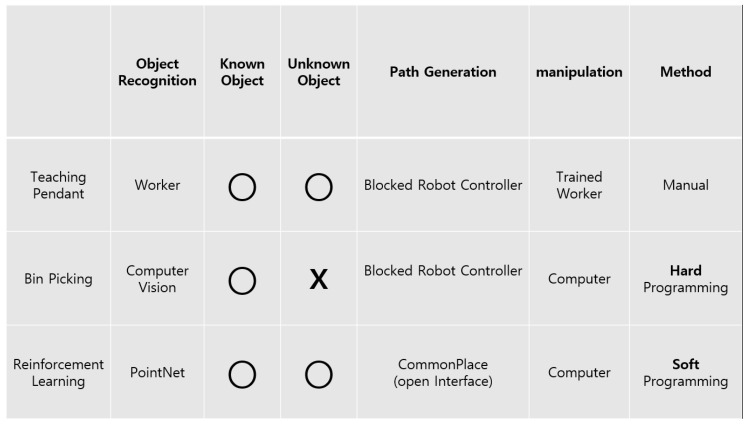
Comparison between Teach-Pendant, Bin-Picking and reinforcement learning (RL).

The most well known and used method for robot arm grasp is teach pendant and bin picking. The comparison of the overall process between them is explained in Figure 13. In the term of automation, RL has much more advantages than other methods. If unknown object is added, teach pendant needs trained worker and bin picking needs to be programmed by programmer manually. But RL can grasp unknown object only by adding CAD file and be trained. Repeatable work done by human could be replaced by our automatic robot gripper system. This system is easily adaptable according to many situation.

(1) Object detection: object recognition task is implemented by using PCD based algorithm. 3D data based approach is the way to utilise more geometric information than 2D images used by 2D image recognition algorithm. From the implementation of the proposed object detection, followings are found. First, in preparation for dataset, if new part comes, several hundred of image data is needed in 2D image recognition. Capturing images is time-consuming and difficult to be automated. PCD is extracted from CAD data which is pre-defined before production line starts to produce the parts. That is CAD data is existed before the part arrived in material handling robot. Simple sampling iteration of point cloud is required for the data collection and is fully automated without additional works for building dataset. Second, in generate variations of part geometry, another benefit to use PCD data is to make many variations of part shapes composed of training dataset. CAD solid kernel has the geometric functions of Boolean operation, extrusion, lofting, and so forth. These functions are used for modifying original CAD shape and building the dataset of its variations. Third, capturing point cloud is the challenges of PCD object detection, 3D camera observes the only one side of part shape which is not sufficient for comparing with whole shape of CAD file. This paper showed the solution of 360-degree rotation table is applied to resolve the issue of capturing PCD in this paper. As for the further possible issue related to capture PCD, other cases to consider are when the object is tilt and when the object shape is complicated.

(2) Reinforcement learning environment: Even though the gripper reaches the optimal grasp position, the time spent searching for the optimal position varies according to the object. For example, in the case of bottle and vase, gripper reaches optimal position easily to bottle rather than vase. It could tell that object’s shape complexity matters and since the bottle has the more constant shape of a cylinder, the gripper arrived at the optimal position earlier. For industrial parts, we should implement more experiments to find which shapes are hard to grasp and other aided methods to increase utility. Due to the limitation of the proposed learning model, reinforcement was conducted step-wise instead of one-step to reach the final goal in a more stable manner. Not using the entire robot arm and using only a gripper for training prevented the learning model from being complex. But it was effective enough for grasping an object in a reasonable position with fine-tuned gripping behaviour. Since there are also many errors in the final gripping operation even in actual robot control, these functions are expected to work effectively in the field as well. For the RL algorithm, PPO is only one of the policy gradient based algorithm. We used SAC which is hybrid algorithm that combines the advantages of policy gradient and Q-Learning but in our case it did not work well. The direction of future studies is changing RL algorithm, for example DQN [27], DDPG [28] and tuning hyper-parameter of PPO and SAC for higher performance.

(3) Robot Programming: Since the 4-DOF robot arm was used in the current implementation, there were some physical limitations. If a robot arm could move, when a higher DOF is used, diverse paths can be formed for a single target coordinate. Therefore, even if there are obstacles interfering to reach the target coordinate, it is possible to avoid these and achieve the goal by using multi paths. However, 4-DOF robot arm can operate perfectly only when there are not any objects around the target due to the physical limitations. In future studies, higher DOF robot arm will be employed to apply RL in more diverse environments. And we should consider precision of robot manipulation. One of the methods for increasing precision at industry 4.0 can be online calibration.

Finally, we should consider applying the GadgetArm System to factories. For example, worker safety (Human-Robot Interaction), change of gripper to a more universal gripper, and the validity of the importing GadgetArm system instead of the current system.

## 6. Conclusions

**Figure 14:**
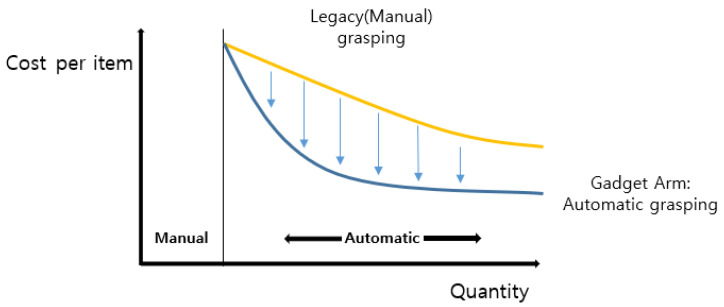
Cost comparison by number of product type.

In this paper, simply combining systems or technology from various fields, it was able to build a system for an automatic robot gripper. Rather than using a passive teach pendant, GadgetArm suggests a method for getting one step closer to achieving Industry 4.0. PointNet for arbitrary object recognition, Unity ml-agents RL for finding optimal grasp position, control the robot control system with 3D printed gripper and connecting these independent processes with one system. As shown in Figure 14, in the range of the quantity appropriate to an automatic process, by adapting GadgetArm, it could lower the cost per item compared to the legacy grasping method. This process can be easily modified according to diverse industry, not only for manufacturing facility but every field that suffers from Pick-n-Place task, or even in the household.

## Figures and Tables

**Table 1:** PPO Hyper-Parameter.

HyperParameter	Value
Batch Size	800
Buffer Size	12,000
Learning Rate	3.0 × $10^{-4}$
Max Steps	500,000
Beta	$5.0\times 10^{-3}$
Epsilon	0.2
Lambda	0.95

## References

[1] Gorecky D., Campos R., Chakravarthy H., Dabelow R., Schlick J., Zühlke D. (2013). Mastering Mass customization—A Concept for advanced, Human-centered assembly. Acad. J. Manuf. Eng..

[2] Bendiksen A., Hager G. A vision-based grasping system for unfamiliar planar objects. Proceedings of the IEEE International Conference on Robotics and Automation.

[3] Perrin D., Masoud O., Smith C.E., Papanikolopoulos N.P. Unknown object grasping using statistical pressure models. Proceedings of the ICRA. Millennium Conference.

[4] Speth J., Morales A., Sanz P.J. Vision-based grasp planning of 3D objects by extending 2D contour based algorithms. Proceedings of the IEEE/RSJ International Conference on Intelligent Robots and Systems.

[5] Wu Z., Song S., Khosla A., Yu F., Zhang L., Tang X., Xiao J. 3d shapenets: A deep representation for volumetric shapes. Proceedings of the IEEE Conference on Computer Vision and Pattern Recognition.

[6] Maturana D., Scherer S. Voxnet: A 3d convolutional neural network for real-time object recognition. Proceedings of the IEEE/RSJ International Conference on Intelligent Robots and Systems (IROS).

[7] Qi C.R., Su H., Mo K., Guibas L.J. Pointnet: Deep learning on point sets for 3d classification and segmentation. Proceedings of the IEEE Conference on Computer Vision and Pattern Recognition.

[8] Wang F., Liang C., Ru C., Cheng H. (2019). An Improved Point Cloud Descriptor for Vision Based Robotic Grasping System. Sensors.

[9] Ji S.Q., Huang M.B., Huang H.P. (2019). Robot intelligent grasp of unknown objects based on multi-sensor information. Sensors.

[10] Erez T., Tassa Y., Todorov E. Simulation tools for model-based robotics: Comparison of bullet, havok, mujoco, ode and physx. Proceedings of the IEEE International Conference on Robotics and Automation (ICRA).

[11] Johns E., Leutenegger S., Davison A.J. Deep learning a grasp function for grasping under gripper pose uncertainty. Proceedings of the IEEE/RSJ International Conference on Intelligent Robots and Systems (IROS).

[12] Plappert M., Andrychowicz M., Ray A., McGrew B., Baker B., Powell G., Schneider J., Tobin J., Chociej M., Welinder P. (2018). Multi-goal reinforcement learning: Challenging robotics environments and request for research. arXiv.

[13] Wang C., Zhang Q., Tian Q., Li S., Wang X., Lane D., Petillot Y., Wang S. (2020). Learning Mobile Manipulation through Deep Reinforcement Learning. Sensors.

[14] Mousavian A., Eppner C., Fox D. 6-dof graspnet: Variational grasp generation for object manipulation. Proceedings of the IEEE International Conference on Computer Vision.

[15] Juliani A., Berges V.P., Vckay E., Gao Y., Henry H., Mattar M., Lange D. (2018). Unity: A general platform for intelligent agents. arXiv.

[16] Reddy P.V.P., Suresh V.V.N.S. (2013). A review on importance of universal gripper in industrial robot applications. Int. J. Mech. Eng. Robot. Res..

[17] Birglen L., Schlicht T. (2018). A statistical review of industrial robotic grippers. Robot. -Comput.-Integr. Manuf..

[18] Du G., Zhang P., Li D. (2015). Online robot calibration based on hybrid sensors using Kalman Filters. Robot. -Comput.-Integr. Manuf..

[19] Kraft D., Ellekilde L.-P., Jørgensen J.A. (2014). Automatic grasp generation and improvement for industrial bin-picking. Gearing Up and Accelerating Cross-fertilization between Academic and Industrial Robotics Research in Europe.

[20] Erdős G., Kardos C., Kemény Z., Kovács A., Váncza J. (2016). Process planning and offline programming for robotic remote laser welding systems. Int. J. Comput. Integr. Manuf..

[21] Weigand J., Volkmann M., Ruskowski M. (2019). Neural Adaptive Control of a Robot Joint Using Secondary Encoders. International Conference on Robotics in Alpe-Adria Danube Region.

[22] Muthusamy R., Huang X., Zweiri Y., Seneviratne L., Gan D. (2020). Neuromorphic Event-Based Slip Detection and suppression in Robotic Grasping and Manipulation. arXiv.

[23] Unity-Technologies, ML-Agents Toolkit Overview, Github, 17 July, 2020. github.com/Unity-Technologies/ml-agents/blob/master/docs/ML-Agents-Overview.md.

[24] Schulman J., Wolski F., Dhariwal P., Radford A., Klimov O. (2017). Proximal policy optimization algorithms. arXiv.

[25] Schulman J., Levine S., Abbeel P., Jordan M., Moritz P. (2015). Trust region policy optimization. Int. Conf. Mach. Learn..

[26] Francis Williams, point-cloud-utils, Github, 7 May, 2020. github.com/fwilliams/point-cloud-utils.

[27] Mnih V., Kavukcuoglu K., Silver D., Graves A., Antonoglou I., Wierstra D., Riedmiller M. (2013). Playing atari with deep reinforcement learning. arXiv.

[28] Lillicrap T.P., Hunt J.J., Pritzel A., Heess N., Erez T., Tassa Y., Silver D., Wierstra D. (2015). Continuous control with deep reinforcement learning. arXiv.

